# Enzymatic Electrochemical/Fluorescent Nanobiosensor for Detection of Small Chemicals

**DOI:** 10.3390/bios13040492

**Published:** 2023-04-19

**Authors:** Hye Kyu Choi, Jinho Yoon

**Affiliations:** 1 Department of Chemistry and Chemical Biology, Rutgers, The State University of New Jersey, Piscataway, NJ 08854, USA; hye.choi@rutgers.edu; 2 Department of Biomedical-Chemical Engineering, The Catholic University of Korea, 43 Jibong-ro, Bucheon-si 14662, Gyeonggi-do, Republic of Korea

**Keywords:** nanobiosensor, enzymatic biosensor, electrochemistry, fluorescence, small molecules

## Abstract

The detection of small molecules has attracted enormous interest in various fields, including the chemical, biological, and healthcare fields. In order to achieve such detection with high accuracy, up to now, various types of biosensors have been developed. Among those biosensors, enzymatic biosensors have shown excellent sensing performances via their highly specific enzymatic reactions with small chemical molecules. As techniques used to implement the sensing function of such enzymatic biosensors, electrochemical and fluorescence techniques have been mostly used for the detection of small molecules because of their advantages. In addition, through the incorporation of nanotechnologies, the detection property of each technique-based enzymatic nanobiosensors can be improved to measure harmful or important small molecules accurately. This review provides interdisciplinary information related to developing enzymatic nanobiosensors for small molecule detection, such as widely used enzymes, target small molecules, and electrochemical/fluorescence techniques. We expect that this review will provide a broad perspective and well-organized roadmap to develop novel electrochemical and fluorescent enzymatic nanobiosensors.

## 1. Introduction

The accurate detection and quantification of small molecules have become increasingly important in various fields, such as chemistry, biology, medicine, and environmental science. Small molecules are ubiquitous in nature and play important roles in various biological processes, including gene expression, metabolism, and cell signaling [1,2]. For instance, neurotransmitters such as dopamine (DA), serotonin, and norepinephrine, are representative small molecules that regulate the mood, behavior, and cognitive function of organisms, and their accurate detection and quantification are essential for understanding neurological disorders such as depression [3,4] and anxiety. In addition, small molecules can be used as biomarkers for various diseases [5]. Therefore, the detection of small molecules can be critical for early disease diagnosis and treatment. For example, glucose is a small molecule that is used as a biomarker for diabetes, and its accurate quantification is necessary for monitoring blood glucose levels and adjusting insulin therapy [6]. Other important molecules, such as prostate-specific antigen (PSA) and carcinoembryonic antigen (CEA), are also used as significant biomarkers for prostate and colon cancer, respectively, in the biomedical field [7,8]. The accurate detection of PSA and CEA can be critical for early cancer diagnosis and treatment. In addition to their biomedical contribution, small molecules can also be used as indicators of environmental pollution and food contamination, which help to ensure public health and safety. Heavy metal ions, such as lead, cadmium, and mercury, are small molecules that can be present in industrial waste, and monitoring the level of these small molecules is necessary to confirm that these contaminants do not affect the environment and harm human health [9]. As described above, small molecules play critical roles in various fields, and their accurate detection and quantification are essential for disease diagnosis, drug discovery, environmental monitoring, and food safety. Therefore, the development of sensitive and selective biosensors for small molecules has the potential to advance these fields and improve human health and safety.

Among the different types of biosensors, enzymatic biosensors have shown improved sensing due to their highly specific enzymatic reactions with small chemical molecules [10]. Enzymatic biosensors typically consist of an enzyme that catalyzes a reaction with the target molecule and a transducer that converts the reaction into a measurable signal [11]. This transducer is developed based on various detection principles, such as electrochemical, optical, or mass-based techniques [12]. Enzymatic biosensors can provide reliable and accurate measurements of various analytes under different conditions. One of the advantages of enzymatic biosensors is their stability under different temperatures and pH levels, making them suitable for use in a variety of settings. Enzymatic biosensors can typically operate at a wide range of temperatures, which allows the biosensors to be used in various applications where the temperature may fluctuate, such as in industrial processes or the outdoors. Additionally, many enzymatic biosensors are stable at room temperature, making them convenient to use and store without the need for special refrigeration or temperature-controlled storage [13,14,15]. Their ability to function reliably under different conditions ensures that they can provide accurate measurements of analytes in various settings, from the laboratory to the field. Compared to non-enzymatic or enzyme-free biosensors, enzymatic biosensors have several advantages, including high specificity and sensitivity, low detection limits, and a wide detection range [16,17]. In addition, enzymatic biosensors can also be tailored to detect specific molecules, making them suitable for a wide range of applications, including clinical diagnosis, environmental monitoring, and food safety. Enzymatic biosensors offer several advantages over antibody- and aptamer-based biosensors. Firstly, enzymes are more cost-effective than antibodies, which can be expensive to purchase [18]. Additionally, in electrochemical biosensors, enzymes capable of undergoing redox reactions can be directly utilized as redox signal probes, whereas antibody-based biosensors typically require additional redox-active molecules such as methylene blue or ferritin [19]. Further, there are limitations to developing biosensors for detecting various small chemicals using aptamers unless a specific aptamer sequence is designed via complex techniques, such as the systematic evolution of ligands by exponential enrichment (SELEX) [20].

To achieve accurate detection of small molecules, electrochemical and fluorescence techniques have been mainly used. Electrochemical techniques are based on the measurement of current or potential changes resulting from the enzymatic reaction, while fluorescence techniques involve the detection of changes in the fluorescence intensity of the reaction product. Both techniques offer high sensitivity, selectivity, and specificity, making them suitable for the detection of a wide range of small molecules [21,22].

The incorporation of nanotechnologies into enzymatic biosensors has further enhanced the performance of enzymatic biosensors, improving their ability to detect harmful or important small molecules with high accuracy. Nanomaterials with unique properties, such as high surface area, conductivity, and catalytic properties, have been utilized to improve the sensitivity, selectivity, and stability of enzymatic biosensors [23]. For example, metal nanoparticles (NPs) and carbon-based nanomaterials (e.g., carbon nanotubes, graphene) have been used to improve the performance of enzymatic biosensors [24,25]. Using these nanomaterials, researchers have increased the surface area available for immobilizing enzymes, created a more favorable microenvironment for enzymatic reactions, and improved signal amplification [26]. The resulting improvements have led to enzymatic biosensors with enhanced sensitivity and selectivity for detecting small molecules. Additionally, the incorporation of nanomaterials has also facilitated the development of hybrid biosensors that combine the advantages of both electrochemical and optical detection techniques, leading to even better detection performance [27,28]. The incorporation of nanotechnologies has opened up new avenues for the development of enzymatic nanobiosensors, allowing researchers to fabricate biosensors that are highly selective and accurate in their detection of small molecules. With ongoing research and development, enzymatic nanobiosensors hold great promise for a wide range of applications in fields such as healthcare, environmental monitoring, and food safety.

**Figure 1:**
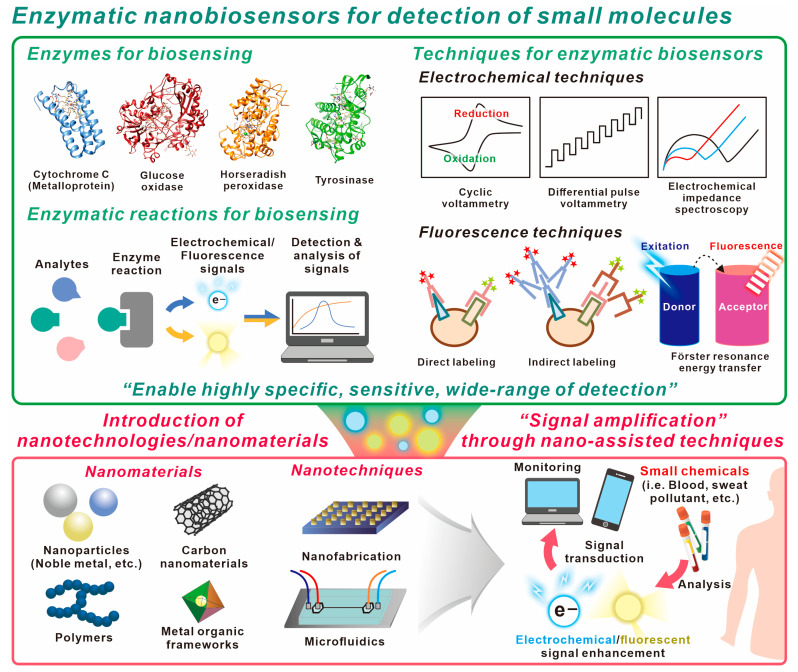
Schematic diagram of enzymatic electrochemical/fluorescent nanobiosensor for detection of small chemicals.

Therefore, this review aims to provide interdisciplinary information on the development of enzymatic nanobiosensors for small molecule detection, including the widely used enzymes, electrochemical/fluorescence techniques, and electrochemical/fluorescent biosensors (Figure 1). Through a well-organized roadmap, this review will offer a broad perspective on the development of novel electrochemical and fluorescent enzymatic nanobiosensors for small molecule detection.

## 2. Components of Enzymatic Biosensors

### 2.1. Enzymes

**Figure 2:**
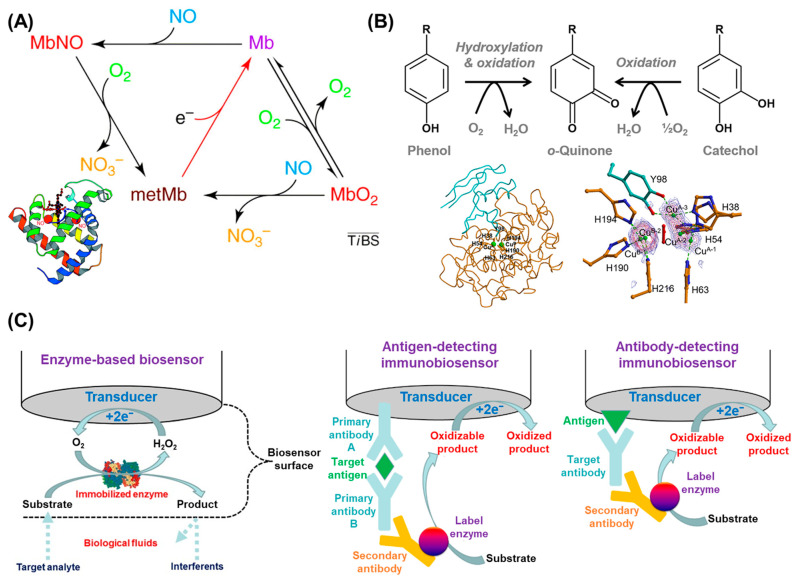
(**A**) Structure of Mb for reaction with NO, and its enzymatic reaction process for detection of NO by regulation of its states, Reprinted with permission from ref. [33]. Copyright © 2001, Elsevier Science Ltd., (**B**) Schematic diagram of enzymatic reactions of the Tyr using its copper ions for conversion of catechol and phenol to *o*-quinone, and its structure containing copper ions, Reprinted with permission from ref. [39]. Copyright © 2018 by the authors, (**C**) Schematic representation of enzyme-based biosensors. Examples of conventional enzymatic biosensors (left) and immune biosensors that employ an enzyme as a labeling component for the indirect detection of a target antigen (middle) or a target antibody (right), Reprinted with permission from ref. [48]. Copyright © 2016 by the authors, Licensee MDPI.

Among various enzymes, metalloenzymes are mainly used in the development of enzymatic biosensors. Since metalloenzymes possess metal ions in their core structure, they can form enzymatic reactions with certain small molecules using these metal ions and have the potential to develop biosensors [29,30]. For example, metalloenzymes have been used to develop biosensors to detect small chemicals that are harmful or require sophisticated measurements in the environment. Representative examples of such enzymes include cytochrome c (Cyt C), myoglobin (Mb), hemoglobin (Hb), and nitrogenase (Nase). Using iron ions in their core, both Mb and Hb act as oxygen carriers. Cyt c possesses an iron ion that can react with hydrogen peroxide (H_2_O_2_) in an enzymatic manner and has been hugely applied to develop H_2_O_2_ biosensors [31,32]. Moreover, as shown in the reaction diagram of Mb in Figure 2A, Mb can also enzymatically react with nitric oxide (NO) [33]. Some examples of enzymatic reactions are discussed in more detail in the next subsection. In addition, Nase reacts with nitrogen and can thus be used to measure nitrogen, which is important in chemistry [34].

In addition, metalloenzymes and some enzymes capable of performing redox reactions are being used to diagnose chemical substances that play important roles in living organisms. Glucose oxidase (GOx) is one of the most widely used enzymes to develop the glucose biosensor. Since glucose plays a large role in diabetes, a metabolic disorder related to the regulation of blood glucose levels, the accurate monitoring of glucose is important in the healthcare field [35]. To achieve this goal, directly monitoring glucose in the blood using an implantable biosensor is more accurate than a traditional blood test. The implantable glucose biosensor uses poly (3,4-ethylenedioxythiophene) (PEDOT) modified carbon fiber (CF) as an electrode [36]. Here, GOx was introduced as an enzymatic probe to detect glucose based on the enzymatic reaction between flavin adenine dinucleotide (FAD) in the active site of GOx and glucose. During this enzymatic reaction, FAD in GOx converts glucose to gluconolactone, and the generated electrons are measured and quantified using the biosensor. GOx is used as a key component not only in the development of the glucose biosensor at the laboratory level but also in the development of commercially available glucose biosensors. Similarly to this, urease, which contains nickel ions, is used to detect urea in healthcare monitoring applications [37].

Recently, in line with stem cell research, some metalloenzymes are also being used for cell differentiation monitoring in the biomedical field. DA is an important chemical neurotransmitter that plays an important role in signal transmission in the nervous system. Horseradish peroxidase (HRP) is an enzyme that is widely used to detect DA [38]. In addition, tyrosinase (Tyr), containing copper ions, can oxidase catechol, a toxic small chemical compound [39]. Using the copper ions in its core, Tyr can react with catechol and convert it to *o*-quinone via oxidation and can also convert phenol to *o*-quinone via hydroxylation and oxidation enzymatic reactions, as shown in Figure 2B. In addition to the enzymes discussed here, there are numerous enzymes that can be used to detect small chemicals (e.g., laccase). Biosensors using such enzymes have high selectivity and specificity, but highly sensitive detection is difficult due to the intrinsic characteristics of biomolecules. In order to address this issue, various nanomaterials and nanotechnologies are being grafted to enzymatic biosensors. For instance, metal nanoparticles have been utilized in enzymatic biosensors for significant enhancement of signal amplification and sensitivity due to their large surface area for higher biorecognition sites and immobilization of biomolecules [40]. In addition, various nanomaterials and nanotechnologies, including 2D nanomaterials (e.g., graphene and carbon dots) and nanofabrication techniques (e.g., electron beam lithography), have been incorporated in enzymatic biosensors to improve sensitivity and stability [41,42,43].

### 2.2. Enzymatic Reactions for Biosensing Applications

In biosensing applications, enzymes are typically immobilized on the surface (electrode or substrate) of the sensor and react with a specific target molecule to produce a detectable signal. The processes of enzyme reactions in biosensing applications typically involve the following steps.

At first, the enzymes recognize and bind to the target molecule, typically through specific binding sites or active sites on the enzyme [44]. Next, the enzymes catalyze chemical reactions with the target molecules that lead to the production of detectable electrochemical/fluorescence signals. Finally, the signal produced by the enzyme reaction is detected and quantified using sensing techniques, such as electrochemical and fluorescence techniques.

To explain in detail, electrochemical biosensing of small molecules can be achieved by immobilizing the enzyme on an electrode surface, where it catalyzes a redox reaction with the target molecule, producing an electrical current that can be measured. For example, the detection of H_2_O_2_ can be achieved through an enzymatic reaction between metalloenzymes possessing iron ions (such as Mb) and the H_2_O_2_ that results in the iron ion of the metalloenzyme being oxidized from a Fe^2+^ state to a Fe^3+^ state accompanying the production of H_2_O via the reduction of H_2_O_2_. During these redox enzymatic reactions, produced or transferred electrons can be measured easily using an electrochemical method. To mimic the efficient enzymatic reaction of metalloenzymes, several artificial metalloenzymes have been developed [45,46]. Likewise, for the detection of glucose, GOx can be immobilized on an electrode surface to catalyze the oxidation of glucose to gluconic acid with H_2_O_2_, producing an electrical current from a redox reaction that is proportional to the glucose concentration [47]. Similar to GOx, several oxidase enzymes (e.g., glutamate oxidase, lactate oxidase, and ascorbate oxidase) have been used as components for electrochemical enzymatic biosensors [48] (Figure 2C). Utilizing an enzyme that produces or modifies a fluorescent molecule upon reaction with the target molecule, fluorescent biosensing can also be conducted. For example, acetylcholinesterase (Ache) can be used to catalyze the hydrolysis of acetylcholine (Ach), producing choline and acetate. This reaction can be coupled to a fluorescent signal using a fluorescent probe that binds to the choline produced in the reaction, producing a measurable fluorescent signal [49]. In addition to the examples mentioned earlier, numerous other enzyme reactions can be used in biosensing applications. For instance, enzymes, such as peroxidase and catalase, can be used to catalyze the oxidation of various substrates using H_2_O_2_ as a co-substrate, producing detectable signals, such as color changes or oxygen gas bubbles [50]. Similarly, enzymes, such as alcohol dehydrogenase and lactate dehydrogenase, can be used to catalyze redox reactions with coenzymes, such as NAD+ and NADH, which can be measured electrochemically or optically [51]. In such reactions, the enzyme facilitates the transfer of electrons between the substrate and the coenzyme, resulting in a change in the redox state of the coenzyme that can be detected. Other enzymes, such as proteases and nucleases, can be used to cleave specific peptide or nucleic acid sequences, generating fragments that can be detected using various techniques, such as mass spectrometry or fluorescence. The enzymes play a crucial role in catalyzing the reactions and facilitating the formation of detectable signals. Using appropriate co-substrates, coenzymes, and reaction conditions can ensure that the redox reactions occur catalytically and produce robust signals. As seen in this section, enzymes have the potential to develop electrochemical or fluorescent biosensors for the detection of small molecules.

### 2.3. Techniques Used in Enzymatic Biosensing

#### 2.3.1. Electrochemical Technique

**Figure 3:**
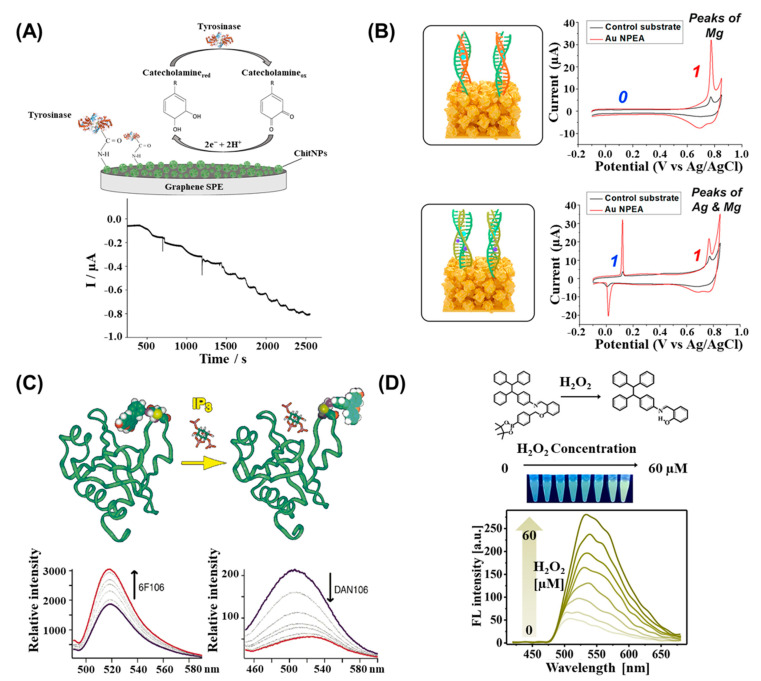
(**A**) Schematic image of amperometric Tyr-based biosensor on ChitNPs for catecholamine detection and its amperometric detection of catecholamine, Reprinted with permission from ref. [56]. Copyright © 2022 by the authors. Licensee MDPI, (**B**) Redox characteristics investigation of metal ions (Mg and Ag) of metal ion–nucleic acid biocomplex using CV, Reprinted with permission from ref. [58]. Copyright © 2022, American Chemical Society, (**C**) A schematic illustration of the structure of IP_3_ complex (top) and Emission spectra of the fluorophore-labeled pleckstrin homology domains in phospholipase C; 6F106 domain (bottom left) and DAN106 domain (bottom right). With an increasing amount of IP_3_, the fluorophore labeled-6F106 domain showed an increase in fluorescence intensity and DAN106 domain showed a decrease in fluorescence intensity, Reprinted with permission from ref. [65]. Copyright © 2002, American Chemical Society, (**D**) Fluorescence spectra with H_2_O_2_ concentrations ranging from 0 to 60 M using H_2_O_2_-triggered “turn-on” fluorescence biosensor, Reprinted with permission from ref. [68]. Copyright © 2019 by the authors.

Among various techniques applicable to developing biosensors, an electrochemical technique is the most actively used technique. The electrochemical method has characteristics required for biosensor development, such as rapid response, inherent miniaturization, high sensitivity, feasibility in practical use, and portability [52,53]. Particularly, the electrochemical technique is advantageous in that many different electrochemical techniques can be applied to develop biosensors that can be used in various ways according to targets and measurement environments [54]. Generally, electrochemical biosensors use electrochemical signal changes from the detection of target molecules using probe molecules, such as the production or removal of electrons or change of impedance. Therefore, to generate electrochemical signal changes, electroactive molecules, such as ferrocene, methylene blue, or the other redox molecules dissolved in the electrolyte, are introduced normally for electrochemical biosensing. Here, metalloenzymes can exclude the additional tagging process of the electroactive molecules. By using its inherent redox properties from metal ions in its structure directly, a metalloenzyme can be considered the best candidate as an electrochemical probe for developing electrochemical biosensors. The amperometry technique, such as amperometric I-T measurement and chronoamperometry, is one of the most broadly used electrochemical techniques to develop metalloenzyme-based electrochemical biosensors using the direct electron transfer reactions between the metalloenzyme and target small molecule [55]. During these electron transfer reactions, the amperometry technique measures the produced or removed electrons directly, and it is normally plotted as stair-like stepwise graph shapes. For instance, the electrochemical Tyr-based biosensor is prepared on a chitosan NPs (ChitNPs)-modified carbon electrode to detect catecholamines composed of catechol and amine side chains [56]. Here, in the presence of oxygen, Tyr catalyzes the oxidization of catecholamine, and the oxidized catecholamine is recovered to its original state by reduction. At this moment, the electrochemical reduction of the oxidized catecholamine can be measured directly by amperometry through the increase in the cathodic signals, as shown in Figure 3A.

In addition, cyclic voltammetry (CV) is also widely utilized to develop electrochemical enzymatic biosensors. Particularly, this technique can investigate the redox activity of metalloenzymes, even in the absence of an enzymatic reaction between metalloenzyme and target small molecules [57]. For example, the redox property of metal ions intercalated in the mismatched nucleic acids was investigated by CV to develop an electrochemical SARS-CoV-2 RNA biosensor for the determination of single-point mutation occurrence [58]. In this study, since certain metal ions can be intercalated in the mismatched nucleic acid sequence to stabilize the overall mismatched nucleic acid structure, the authors designed the probe DNA sequence capable of hybridizing with target SARS-CoV-2 RNA and forming intended mismatched sequences. Using this metal ion–nucleic acid biocomplex, the authors successfully determined the single-point mutation occurrence (Figure 3B). In the presence of pure SARS-CoV-2 RNA, only one redox peak pair was measured, but two different redox peak pairs were measured when the mutation occurred due to the formation of additional metal ion–nucleic acid biocomplexes. In addition, differential pulse voltammetry (DPV) is a broadly used electrochemical technique. In this technique, the measured current difference at two points immediately before and after the potential applied to the biosensor rises is expressed as a graph of voltage [59]. Since this technique measures the current difference obtained before and after the potential rises, it is less disturbed by current measurement disturbances such as electrical double layers. Using DPV, the enzymatic glutathione biosensor was developed based on glutathione peroxidase and graphene oxide (GO) [60]. The electrochemical technique can also be used for the development of electrochemical biosensors using redox-free proteins, enzymes, and antibodies. Electrochemical impedance spectroscopy (EIS) measures the electrical resistance or capacitance of the layers formed through the capture of targets such as antigens by probes like an antibody, so it does not require the inherent redox properties from biomolecules or target molecules [61,62].

However, despite the many advantages of electrochemical techniques, it may sometimes be difficult to sensitively and reliably measure the redox signals derived only from biomolecular reactions because of the inherent limitations of biomolecules. Further, to achieve high sensitivity and selectivity, electrochemical biosensors inevitably require high conductivity to measure the redox signals produced by target detection. To grant sufficient conductivity to biosensors and to solve the intrinsic limitations of biomolecules (e.g., low signal production and low stability), with the introduction of various nanomaterials and nanotechnologies to enzymatic electrochemical biosensors, the studies for developing functional electrodes with high conductivity or the increase in electron transfer of the enzymatic reactions are becoming parallel.

#### 2.3.2. Fluorescence Technique

The fluorescence technique is a widely used technique for developing enzymatic biosensors because it can provide sensitive and specific detection of biomolecules. The basic principle of fluorescence biosensing is that when a molecule is excited by light of a certain wavelength, it absorbs the energy and emits light at a longer wavelength [63]. In a fluorescent biosensor, the target molecule is labeled with a fluorescent molecule or a fluorescent molecule is labeled with the sensing probe conversely, that can be excited by a specific wavelength of light. When the labeled molecule interacts with the biosensor, it changes the local environment of the fluorescent molecule, which alters its emission properties. By measuring the change in fluorescence, the presence and concentration of the target molecule can be detected. To design and develop the fluorescent biosensor, several approaches can be applied. First, a direct labeling method can be utilized to detect the target molecule using the fluorescence technique. To detect ions or small molecules, single directly labeled proteins or peptide-based biosensors have been developed [64]. For example, D-myo-inositol-1,4,5-trisphosphate (IP_3_), an important signaling molecule that modulates cellular Ca^2+^ concentrations, can be detected by signal transduction from a cysteine mutation and alkylation of the active site (pleckstrin homology domain) of phospholipase C, such as 6F106 and DAN106 domains [65] (Figure 3C). The direct labeling method allows simple and straightforward sensing, but it can be challenging to label the target molecule without any damage or effect on the function and structure of the target molecules [66,67].

The indirect labeling method has been used to address the limitations of direct labeling fluorescence methods. In indirect labeling, a probe molecule that is labeled with a fluorescent molecule can specifically bind to the target molecule, and the fluorescence signal is altered from the conjugation of the probe and target molecules. For instance, amantadine can be quantitatively detected using an indirect competitive enzyme-linked immunosorbent assay [68]. Here, amantadine and ovalbumin are conjugated as a coating antigen and coated on a 96-well plate. GOx can also be employed as an enzyme label for immunoassays for GOx/glucose-mediated H_2_O_2_ production, resulting in a fluorescence response at 540 nm (Figure 3D). The Förster resonance energy transfer (FRET) is a phenomenon in which energy is transferred between two fluorescent molecules when they are in close distance proximity [69]. In a FRET-based biosensor, the biosensor contains two fluorescent molecules, a donor and an acceptor. The donor is excited by light and transfers its energy to the acceptor if it is in close proximity. The distance between the two fluorescent molecules changes when the target molecule binds to the biosensor, which alters the FRET signal. Several fluorescence techniques have also been utilized in biosensors, such as luminescence resonance energy transfer, photoluminescence, and electrochemiluminescence [70,71,72]. Overall, fluorescent biosensors have been used in many applications, including the detection of biomarkers for diseases, the monitoring of enzyme activity, and the study of protein–protein interactions. Fluorescent biosensors are versatile and sensitive tools that can provide real-time, non-invasive detection of biomolecules in complex biological environments.

While fluorescent biosensors offer many advantages over traditional enzymatic biosensors, such as high sensitivity, selectivity, and the ability to detect multiple analytes simultaneously, there are also some limitations to their use. One major limitation of fluorescent enzymatic biosensors is the requirement for an external excitation source, such as a laser or UV light, to produce a measurable signal. The necessity of an external source can limit the portability and ease of use of the biosensor in further applications [73]. In addition, the use of external excitation sources can lead to the production of background fluorescence from biological samples, which can interfere with the accuracy of the measurements. Another limitation of fluorescent enzymatic biosensors is the photobleaching of fluorescent dyes over time. The unexpected photobleaching can reduce the sensitivity and stability of the biosensor, making it less reliable over extended periods of time [74]. The bleaching process can be accelerated by exposure to light, temperature changes, and other environmental factors. In addition, the design and optimization of fluorescent enzymatic biosensors can be a complex and time-consuming process [75]. The choice of the fluorescent molecule, the method of attachment to the enzyme or substrate, and the selection of appropriate detection parameters all play important roles in determining the sensitivity and specificity of the biosensor. The optimization of the parameters requires extensive experimentation and validation, adding to the cost and time required to develop and manufacture the biosensor. Despite these limitations, fluorescent enzymatic biosensors remain a valuable tool for a wide range of applications, including medical diagnostics, environmental monitoring, and food safety testing. Current research and development in the field are focused on improving the sensitivity, stability, and ease of use of these biosensors to make them more practical and accessible for a variety of applications with the incorporation of nanomaterials and nanotechnologies.

## 3. Enzymatic Electrochemical Nanobiosensors

**Figure 4:**
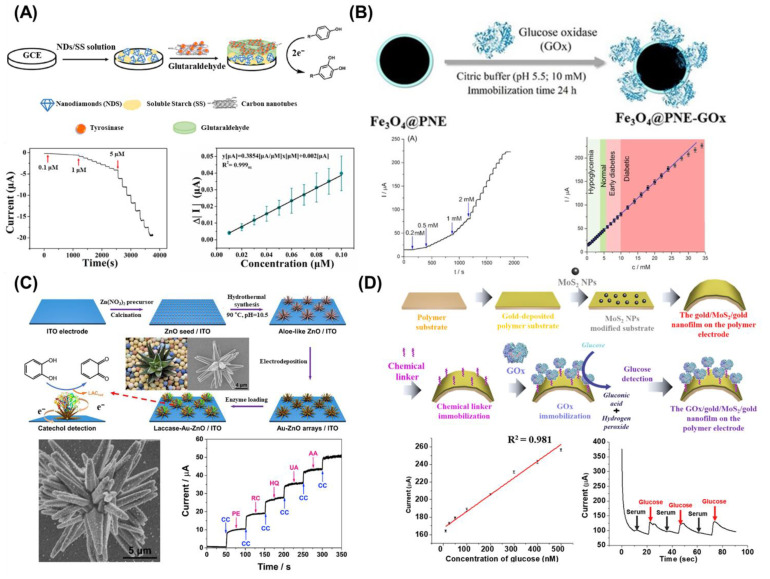
(**A**) Schematic illustration of the fabrication of nanobiosensor composed of Tyr, ND, and CNT, and electrochemical phenol detection results, Reprinted with permission from ref. [79]. Copyright © 2022 by the authors, (**B**) Scheme for encapsulation of the MNP by PNE and immobilization of GOx, and amperometric response and linear response plot for glucose detection in the presence of a different concentration of the glucose, Reprinted with permission from ref. [84]. Copyright © 2022 Elsevier B.V., (**C**) Schematic diagram of catechol nanobiosensor fabrication by using Au-ZnO nanocrystal arrays and Laccase, scanning electron microscopy (SEM) image of Au-ZnO nanocrystal, and its selective electrochemical catechol (CC) detection property, Reprinted with permission from ref. [87]. Copyright © 2020 Elsevier B.V., (**D**) Schematic image of flexible GOx/Au/MoS_2_/Au nanofilm-based nanobiosensor and electrochemical results for glucose detection with high linearity prepared in the serum sample, Reprinted with permission from ref. [89]. Copyright © 2019 Elsevier B.V.

As discussed above, numerous biosensors have been developed so far using various enzymes, particularly metalloenzymes, and electrochemical techniques. These enzymatic biosensors have difficulty in highly sensitive diagnostics due to the intrinsic characteristics of biomolecules, such as low activity and stability, and therefore have limitations in diagnostics in real samples or harsh conditions. To overcome this limitation, in recent years, with the introduction of nanomaterials or nanotechnologies, nanobiosensors that measure targets in real samples or real environments with high sensitivity are being researched [76,77]. In terms of electrochemical biosensors, nanomaterials and nanotechnologies are being studied to accelerate enzymatic reactions and promote electron transfer or fabricate functional conductive electrodes [25,78]. In one study, carbon nanomaterials and nanodiamonds (ND) were employed to develop the Tyr-based amperometric nanobiosensor for detecting small pollutant chemicals (Figure 4A) [79]. Here, as the electrode materials of an enzymatic electrochemical nanobiosensor, carbon nanotubes (CNTs), NDs, and starch were used to develop a nanobiocomposite on a glassy carbon electrode (GCE). To fabricate this, NDs combined with soluble starch (ND-SS) were prepared on the GCE first, and then, the Tyr-trapped CNTs were anchored on the ND-SS using glutaraldehyde (Glu). The developed nanobiocomposite provided enhanced load capacity for Tyr, improved electron transfer efficacy from CNTs, and provided the biocompatibility for Tyr via the introduction of ND-SS. Consequently, the redox signals from this nanobiocomposite-based nanobiosensor during the detection of small pollutant chemicals (phenolic compounds) exhibited sufficiently enhanced signals in various real water samples with a 2.9 nM limit of detection (LoD) and long-term activity in an amperometric manner. In addition to this study, there are some recent studies developing enzymatic nanobiosensors by introducing nanomaterials to enzymes. For instance, a nanocomposite composed of an AuNP and titanium disulfide (TiS_2_) nanosheet (NS) was developed and combined with uricase to develop an amperometric uric acid nanobiosensor. Due to the synergistic effects of the AuNP and TiS_2_ NS for increasing the redox signals produced from the enzymatic reaction between uricase and uric acid, the developed nanobiosensor successfully detected uric acid prepared with interfering molecules, such as ascorbic acid, urea, and glucose, and detected uric acid dissolved in commercial human serum [80]. In another study, gold (Au) and platinum (Pt)-decorated CNTs were proposed as the nanocomposite for the immobilization of cholesterol oxidase (COx) to develop an enzymatic electrochemical cholesterol nanobiosensor [81]. In addition to the metallic nanocomposite, other materials, such as organic materials, are also being incorporated with metallic nanomaterials to develop enzymatic nanobiosensors. In one study, polyaniline (PANI), a conducting polymer, was combined with titanium dioxide (PANI@TiO_2_) and prepared on an indium tin oxide (ITO) electrode using electrophoretic deposition to provide enhanced electron transfer kinetics for xanthine oxidase (XOs) immobilized on the PANI@TiO_2_ electrode [82]. Using the enzymatic activity of XOs for the detection of xanthine (Xn), Xn was detected sensitively with a 100 nM LoD and rapid response (within 10 s) using the DPV technique. Additionally, various functional nanocomposite-based enzymatic nanobiosensors have been reported, such as the use of magnetic NPs (MNPs) [83,84]. For instance, the MNP, Iron (III) oxide (Fe_3_O_4_), encapsulated by polynorepinephrine (PNE), was developed and combined with GOx to develop an enzymatic glucose nanobiosensor operable with a smartphone (Figure 4B) [84]. Due to the encapsulation of the MNPs by PNE, PNE increased the enzyme immobilization efficiency doubly compared to bare MNPs for achieving improved enzymatic activity of GOx for glucose detection. The developed nanobiosensor successfully and rapidly detected glucose in human serum, blood, and commercially available glucose solutions using a smartphone that supports the applicability of this nanobiosensor for point-of-care testing (POCT) in broad industries. Moreover, the ability to recollect MNPs by a magnet can be further utilized in biomedical applications.

In addition to nanomaterial-assisted enzymatic electrochemical nanobiosensors, through the development of electrodes with improved conductivity via nanotechnologies, various novel enzymatic electrochemical nanobiosensors are also being studied. Nanotechnologies, such as lithography, microelectromechanical systems (MEMSs), and nanoelectromechanical system (NEMS), are being used to develop conductive functional electrodes [85,86]. For instance, the enzymatic electrochemical glucose nanobiosensor was developed by combining reduced GO (rGO), GOx, a microfluidics chip, and a 3D-printed paper electrode [86]. This nanobiosensor measured the glucose in human sweat and blood via the enzymatic catalytic reaction of GOx with glucose, and the utilized nanotechnologies offered high conductivity for this enzymatic reaction and large surface areas for GOx immobilization. This study suggested one approach for fabricating disposable nanobiosensors. In addition to this, there was a study that used micro/nanoarrays composed of Au and zinc oxide (Au-ZnO) nanocrystals developed as an electrode for the enzymatic detection of catechol (Figure 4C) [87]. In this study, to fabricate the Au-ZnO nanocrystals, the zinc nitrate (Zn(NO_3_)_2_) precursor was prepared on the ITO electrode, and then, the ZnO nanocrystals were developed via a hydrothermal reaction. Next, through the electrodeposition of Au on the ZnO nanocrystals, the Au-ZnO nanocrystals were finally developed on the ITO electrode (Figure 4C), and laccase was immobilized on the Au-ZnO nanocrystals as a sensing probe enzyme. The developed Au-ZnO nanocrystal-assisted nanobiosensor detected catechol selectively and sensitively using the amperometry technique and also accurately measured catechol in tap water and lake water. Additionally, along with the development of smart devices, such nanotechnologies have the tremendous potential to develop flexible or wearable electrochemical nanobiosensors, which have received attention recently in the field of biosensors, particularly for the development of flexible or implementable conductive electrodes [88]. The development of wearable, flexible, or implementable nanobiosensors can enable the measurement of small chemicals directly in the body instead of using an in vitro solution. For the easy and simple fabrication of flexible electrodes as enzymatic nanobiosensors, one research group developed a flexible enzymatic electrochemical nanobiosensor composed of molybdenum disulfide (MoS_2_) NPs, an Au nanolayer, and GOx to detect glucose (Figure 4D) [89]. Here, instead of relatively complex techniques, such as lithography, electrochemical deposition, and the sputtering technique were employed to fabricate the sandwiched structural nanofilm (Au/MoS_2_/Au) on the flexible polymer electrode capable of providing a large surface area and facilitating electron transfer with sufficient bendability with GOx immobilized on the Au/MoS_2_/Au nanofilm via a chemical linker. The developed flexible nanobiosensor detected glucose with nanomolar sensitivity and high selectivity while retaining its flexibility. In another example, a bendable electrode composed of the silver nanowire (AgNW) and electropolymerized polymers was developed to be used for the noninvasive detection of lactate in human sweat [90]. In addition to this, the 3D printing technique, which has recently attracted attention in various scientific fields, is also being applied to develop novel enzymatic electrochemical nanobiosensors [91]. As discussed in this section, numerous enzymatic electrochemical nanobiosensors have been studied through the introduction of nanomaterials or nanotechnologies with functional enzymes to detect small chemicals. Such studies are expected to contribute to the development of enzymatic electrochemical nanobiosensors for POCT application in a simple and rapid detection manner in the near future.

## 4. Enzymatic Fluorescent Nanobiosensors

**Figure 5:**
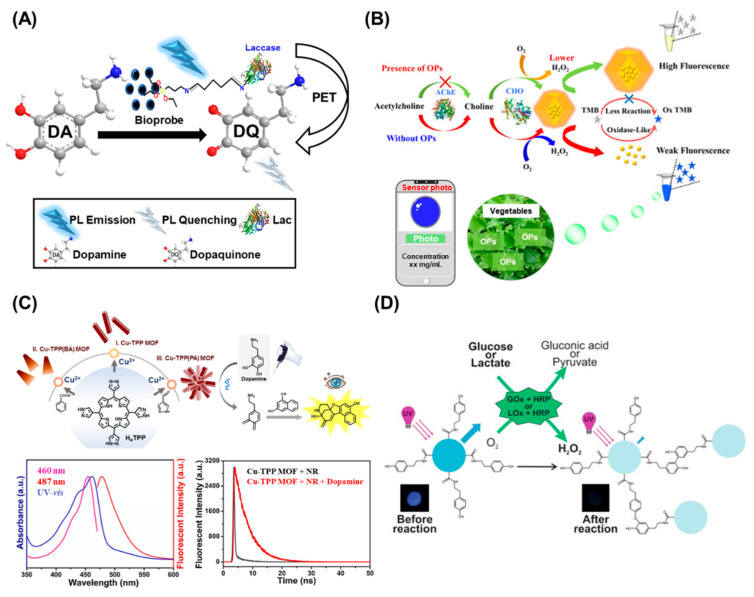
(**A**) Schematic representation for the plausible mechanism of photoluminescence quenching of bioprobe by DA, Reprinted with permission from ref. [92]. Copyright © 2021, Elsevier B.V., (**B**) Schematic Diagram of the Mechanism for the Detection of OPs, Reprinted with permission from ref. [99]. Copyright © 2021, American Chemical Society, (**C**) Schematic illustration of the sensing process of Cu-TPP MOFs toward DA (top), UV vis absorption spectra at 460 nm, fluorescence excitation spectra at 460 nm, and emission spectra at 487 nm for the final product aqueous solution (bottom left), and time-resolved photoluminescence emission decay spectra of Cu-TPP MOF with and without DA, Reprinted with permission from ref. [105]. Copyright © 2021 Elsevier B.V., and (**D**) Illustration of the proposed mechanism of carbon dots fluorescence quenching when reacting with glucose in the presence of GOx and HRP or lactate in the presence of lactate oxidase and HRP., Reprinted with permission from ref. [110]. Copyright © 2020 Elsevier B.V.

Similar to enzymatic electrochemical nanobiosensors, enzymatic fluorescent nanobiosensors have gained significant attention in recent decades due to their high sensitivity and selectivity, as well as their capability to monitor analytes or targets in real time. With the integration of nanomaterials or nanotechnologies in fluorescent biosensors, the sensitive and selective detection of analytes or targets is allowed, while enzymatic reactions enhance the specificity and accuracy of the detection. One of the general approaches to developing enzymatic fluorescent nanobiosensors is the immobilization of fluorescent NPs on specific enzymes, which can result in fluorescent excitation through an enzymatic reaction between targets and enzymes. One study utilized silica-functionalized carbon dots to achieve multi-color imaging detection of DA [92]. In this study, a bioprobe was fabricated using silica-functionalized carbon dot-immobilized laccase enzyme, and the bioprobe was coated on optical fibers via the dip-coating method (Figure 5A). In the presence of DA, since the laccase enzyme contained in the bioprobe facilitates an oxidation reaction in which the DA is oxidized, DA is transformed into dopaquinone. Then, photoinduced electron transfer 12 occurs between the bioprobe and dopaquinone, with the carbon dots acting as electron donors and dopaquinone as acceptors. As a result, photoluminescence quenching of the bioprobe occurs, and DA can be quantitatively detected with a linear range of 0 to 30 μM. In addition, the developed bioprobe showed selectivity to DA only, as shown by the highest quenching efficiency compared with other interferants, such as metal ions, amino acids, macromolecules, and neurotransmitters. Among the several nanomaterials, the metal–organic framework (MOF) has attracted considerable attention from researchers in the biosensor field [93,94]. The MOF has unique properties and advantages in biosensing compared with other nanomaterials, such as (i) enabling target binding with probe molecules via π-π stacking [95], (ii) high surface area with porosity [96], (iii) easy functionalization and tailoring by controlling the ratio of metal ions in the MOF [97], and (iv) stability [98]. For example, organophosphorus pesticides (OPs), which can bind with AChe resulting in the breakdown of neurotransmitters, were detected using a MOF-applied nanobiosensor [99] (Figure 5B). Herein, the Au nanocluster (AuNC)-modified zeolite-like imidazole framework structure (ZIF) composite (AuNCs@ZIF) was developed as fluorescence material. The AuNCs@ZIF emitted intense fluorescence because ZIF-8 restricted the movement of the AuNCs, inhibited the nonradiative decay, and activated the radiation channel. The combination of AChE and choline oxidase (CHO) can hydrolyze ACh to generate H_2_O_2_, which can degrade the structure of ZIF and diminish the fluorescence of AuNCs@ZIF. Furthermore, the OPs can limit the activity of AChE, resulting in a decrease in the generation of H_2_O_2_, a weakening of ZIF degradation, and a slow fluorescence recovery. As a result, a “turn-on” fluorescence mode was developed, and the detection of OPs was achieved with a 0.4 μg/L LoD. As briefly mentioned in Section 2.2, artificial enzymes composed of nanomaterials that can act as enzymes in the presence of an analyte have been developed. These artificial enzymes are called nanozymes, and numerous studies reported several types of nanozymes. For example, the MOF, which is fabricated by modulating synthetic methods to combine the advantages of natural enzymes and nanomaterials, has a similar catalytic function to natural enzymes [100]. The MOFs are very flexible to biomimetic design and can accommodate a variety of enzymes. For instance, the “armor-like” exoskeleton of MOFs around enzymes can transport small molecules selectively while guaranteeing the stability of enzymes [101]. Since the nanozymes composed of MOFs can be easily tuned and can detect the target small molecules, several nanozymes that mimic oxidase, peroxidase, catalase, and hydrolase have been developed [102,103,104]. As an example, the flower cluster morphology of dicopper (II) complexes (Cu-TPP MOF) was synthesized to detect DA (Figure 5C) [105]. Significantly, by imitating the active sites of a binuclear Cu (II) metal center coordinated by six nitrogen-containing coordination units in natural catechol oxidase, the Cu-TPP MOF demonstrated a high potential for simulating natural biological enzymes. Additionally, applying the flower cluster morphology, the surface area of Cu-TPP increased, which resulted in a change of contact between the DA and the substrate that was significantly enhanced. Cu-TPP MOF was utilized as a mimic of catechol oxidase in the presence of H_2_O_2_ to catalyze the oxidation of DA, producing the equivalent catechol derivative, o-quinone intermediate. Dihydroxynaphthalene was then used as an indicator to react with o-quinone, and a fluorescent signal was promptly generated. Eventually, the DA detection using Cu-TPP MOF with a 2.5 nM LoD was achieved. Similarly, metal oxide- or carbon-based NPs have been utilized as nanozymes [106]. For instance, mercury metal was detected using the peroxidase-like property of polyvinylpyrrolidone Ag NPs [107]. The catalytic activity of synthesized Ag NPs oxidized the o-phenylenediamine (non-fluorescence reagent) to 2,3-diaminophenazine, a high-fluorescence reaction product. In addition, since only mercury (II) ions among the heavy metals inhibited the catalytic reaction, the fluorescence intensity was quenched in the presence of mercury (II) ions. Accordingly, mercury (II) ions were detected with a linear range of 20–2000 nM and an 8.9 nM LoD.

In addition, through the integration of microfluidic technology and enzymatic reactions, microfluidic-assisted fluorescent enzymatic biosensors have been reported to detect specific molecules or analytes in a sample [108]. The enzymatic reactions in microfluidic systems can be facilitated by immobilizing the enzyme onto a surface, such as a microfluidic channel wall or a microbead, or by incorporating the enzyme into the fluid stream. Once the enzyme is immobilized or introduced into the fluid stream, the sample is introduced into the microfluidic system, and the enzyme catalyzes a reaction with the target analyte [109]. With the incorporation of nanomaterials in microfluidic devices, various enzymatic fluorescent biosensors have been developed and offer faster response times, higher sensitivity, and the ability to analyze small sample volumes. For instance, a 3D paper-based analytical device composed of carbon dots was developed to analyze saliva samples (Figure 5D) [110]. To detect glucose and lactate, fluorescence from the carbon dots was used to quantify the H_2_O_2_ generated during the enzymatic oxidation of the analyte. The carbon dot dispersion had a blue emission under UV light in the absence of H_2_O_2_, but the intensity was reduced in the presence of H_2_O_2_ and HRP, allowing the measurement of glucose and lactate via the quenching of fluorescence.

In summary, enzymatic fluorescent nanobiosensors have emerged as a promising tool for the sensitive and selective detection of analytes or targets in real time. The integration of NPs or nanotechnologies into fluorescent biosensors has allowed for sensitive and selective detection, while enzymatic reactions have enhanced the specificity and accuracy of detection. The immobilization of fluorescent NPs to specific enzymes is a general approach used to develop enzymatic fluorescent nanobiosensors. Nanomaterials, such as the MOF, have unique properties and advantages in biosensing. In addition, artificial enzymes composed of nanomaterials, nanozymes, have been developed, which can mimic natural enzymes and possess a high potential for simulating natural biological enzymes. Nanotechnologies, such as microfluidics with NPs, enable the fabrication of novel fluorescent enzymatic biosensors. These advances in the development of enzymatic fluorescent nanobiosensors will provide a promising platform for the sensitive and selective detection of various analytes or targets for various applications in areas such as medical diagnostics, environmental monitoring, and food safety.

## 5. Conclusions and Future Perspectives

The precise detection and quantification of small molecules serve a vital role in various disciplines, including healthcare, environmental monitoring, and food safety. Enzymatic biosensors have shown exceptionally high sensing capabilities, which can be attributed to the specificity with which enzymes react with relatively small chemical molecules. In addition, enzymatic biosensors have various advantages over enzyme-free or non-enzymatic biosensors, including high specificity and sensitivity, low detection limits, and a broad detection range. To develop enzymatic biosensors, electrochemical and fluorescence techniques have been used to detect small molecules, offering high sensitivity, selectivity, and specificity. Moreover, through the incorporation of nanotechnologies, enzymatic biosensors have seen considerable performance improvements, which have led to the development of enzymatic nanobiosensors that have enhanced sensitivity and selectivity for the detection of small molecules [111,112,113,114,115,116] (Table 1). The development of enzymatic nanobiosensors has a wide range of potential applications, including but not limited to medicine, the monitoring of the environment, and food safety. In this review, interdisciplinary information on the development of enzymatic nanobiosensors for small molecule detection, including commonly used enzymes, electrochemical/fluorescence approaches, and electrochemical/fluorescent biosensors, was discussed. In addition, this review provided a comprehensive overview of the development of innovative electrochemical and fluorescent enzymatic nanobiosensors for the detection of small molecules.

While enzymatic nanobiosensors have shown great promise for the detection of small molecules, there are still some limitations that need to be addressed. One major challenge is the stability and reproducibility of these nanobiosensors, particularly when they are exposed to complex biological or environmental samples [117]. Another limitation is the potential interference from other molecules in the sample, which can affect the accuracy and specificity of the nanobiosensors [118]. Additionally, the integration of nanomaterials into enzymatic biosensors normally requires a complex and expensive process, requiring specialized equipment and expertise. Molecularly imprinted polymers (MIPs) have the potential to overcome the limitations of enzymatic biosensors and provide an alternative means of analysis. Compared to enzyme-based devices, MIPs offer superior chemical stability, cost-effectiveness, and ease of fabrication, making them an attractive option for various analytical applications [119,120]. Despite these challenges, recent studies and development in the field of enzymatic nanobiosensors promise to overcome these limitations and improve their performance. Advancements in nanomaterial synthesis and fabrication techniques, as well as the development of new enzyme technologies, are likely to lead to the development of more stable, selective, and sensitive nanobiosensors in the future. Additionally, the integration of machine learning and artificial intelligence may help to overcome some of the challenges associated with interference from other molecules and improve the accuracy of small molecule detection [121]. Moreover, the integration of different nanotechnologies, such as nanofabrication and microfluidics, can facilitate the fabrication of advanced enzymatic nanobiosensors. This integration can enhance the performance and enable multiplex detection of small molecules [58,122]. The progress in the development of enzymatic nanobiosensors will have a significant impact on various fields and lead to the improvement of human health and safety.

## Figures and Tables

**Table 1:** The representative electrochemical/fluorescent enzymatic nanobiosensors illustrated in this review and the other enyzamtic nanobiosensors using Surface-enhanced Raman spectroscopy (SERS), Surface plasmon resonance (SPR), and colorimetric techniques.

Sensing Technique	Enzymatic Reaction	Nano-Assistance	Target	LoD	Ref.
Electrochemical	Tyrosinase reaction	Carbon nanotubes Nanodiamonds	Phenolic compounds	2.9 nM	[79]
	Cholesterol oxidase reaction	Carbon nanotubes	Cholesterol	0.5 µM	[81]
	Glucose oxidase reaction	Polynorepinephrine grafted on magnetite nanoparticles	Glucose	6.1 µM	[84]
	Laccase reaction	Gold–Zinc oxide micro/nanoarrays	Catechol	25 nM	[87]
	Glucose oxidase reaction	Glucose oxidase/Gold/ Molybdenum disulfide/ Gold nanofilm	Glucose	10 nM	[89]
Fluorescent	Laccase reaction	Silica-functionalized carbon dots	Dopamine	41.2 nM	[92]
	Acetylcholinesterase and choline oxidase reaction	Au nanoclusters modified zeolite-like imidazole framework	Organophosphorus pesticides	1.79 nM	[99]
	Catechol oxidase reaction	Pyrazolate-based porphyrinic metal–organic framework	Dopamine	2.5 nM	[105]
	Peroxidase reaction	Polyvinylpyrrolidone stabilized silver nanoparticles	Mercury (II) ion	8.9 nM	[107]
	Glucose oxidase and lactate oxidase reaction	Carbon dots	Glucose Lactate	2.6 µM 0.8 µM	[110]
SERS	Peroxidase- mimicking reaction	Silver nanoparticles/metal organic framework	Cholesterol	0.36 µM	[111]
	Glucose oxidase- like reaction	Silver/gold nanoparticles	Glucose	50 nM	[112]
SPR	Glucose oxidase oxidation	Polystyrene nanoparticle with Manganese dioxide	Glucose	3.1 pM	[113]
	Acetylcholinesterase reaction	Molybdenum disulfide/gold nanoparticle multicore fiber	Acetylcholine	14.28 µM	[114]
Colorimetric	Uricase, glucose oxidase, choline oxidase reaction	Magnetic nanoparticles	Uric acid Glucose Choline	0.34 µM 0.59 µM 0.20 µM	[115]
	Glucose oxidase reaction	Acrylamide based-copolymer hydrogel	H_2_O_2_ Glucose	8.9 µM 1.6 mM	[116]

## Data Availability

Not applicable.
